# Epstein-Barr Viruses (EBVs) Deficient in EBV-Encoded RNAs Have Higher Levels of Latent Membrane Protein 2 RNA Expression in Lymphoblastoid Cell Lines and Efficiently Establish Persistent Infections in Humanized Mice

**DOI:** 10.1128/JVI.01873-15

**Published:** 2015-09-02

**Authors:** Goran Gregorovic, Elizabeth A. Boulden, Rachel Bosshard, Claudio Elgueta Karstegl, Rebecca Skalsky, Bryan R. Cullen, Cornelia Gujer, Patrick Rämer, Christian Münz, Paul J. Farrell

**Affiliations:** a Section of Virology, Imperial College, London, United Kingdom; b Department of Molecular Genetics and Microbiology and Center for Virology, Duke University Medical Center, Durham, North Carolina, USA; c Viral Immunobiology, Institute for Experimental Immunology, University of Zürich, Switzerland

## Abstract

Functions of Epstein-Barr virus (EBV)-encoded RNAs (EBERs) were tested in lymphoblastoid cell lines containing EBER mutants of EBV. Binding of EBER1 to ribosomal protein L22 (RPL22) was confirmed. Deletion of EBER1 or EBER2 correlated with increased levels of cytoplasmic EBV LMP2 RNA and with small effects on specific cellular microRNA (miRNA) levels, but protein levels of LMP1 and LMP2A were not affected. Wild-type EBV and EBER deletion EBV had approximately equal abilities to infect immunodeficient mice reconstituted with a human hematopoietic system.

## TEXT

Epstein-Barr virus (EBV)-encoded RNAs (EBERs) are abundant viral noncoding RNAs in EBV-transformed lymphoblastoid cell lines (LCLs). We previously identified cell genes whose expression in EBV LCLs correlates with deletion of EBER1 or EBER2 (1); here, we used LCLs to test various mechanisms that have been proposed for EBER function.

### Binding of EBER1 to ribosomal protein L22 (RPL22) in LCLs is confirmed, but no effect of EBER1 on p53 protein level was detected.

**FIG 1:**
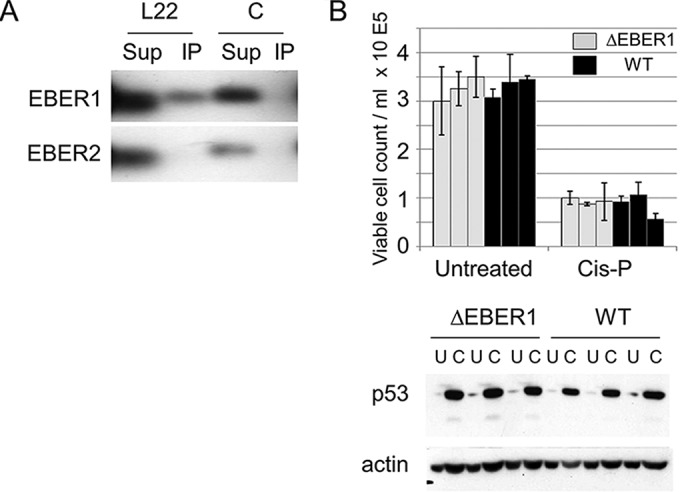
(A) Flag-tagged RPL22 (L22) or negative-control (C) (Flag-tagged Schlafen) proteins were expressed by Neon transfection in LCL cells containing B95-8 BAC EBV. Cell extracts were incubated with M2 anti-FLAG antibody (Sigma-Aldrich) bound to protein G Sepharose beads. RNA was extracted from the beads (using immunoprecipitation [IP]) and the unbound supernatant (Sup) and tested for EBER by Northern blotting. (B) Cells from three independent ΔEBER1 LCLs (light gray bars) or wt EBV LCLs (WT; black bars) were treated with 20 μg/ml cisplatin (C) in triplicate or were left untreated (U) as a control. For each sample, 2 ml of cell suspension (at 2.5 × 10^5^ viable cells/ml, determined by trypan blue exclusion) was set up per well in 6-well plates with or without cisplatin as indicated. The number of viable cells (excluding trypan blue) was determined after 16 h, and p53 protein was detected by Western blotting of cell extracts made at the same time point. DO1 antibody (Santa Cruz) was used for detection of p53 by Western blotting. Actin was used as a loading control on the Western blots.

EBER1 can bind to RPL22 (2), and studies on RPL22 knockout mice (3, 4) showed a p53-dependent defect in pro-B cells (4). The effect is thought to involve RPL22 binding to p53 mRNA, reducing translation of p53 (5). It is possible that EBER1 could modulate this pathway, although EBV infects mature B cells, in which the RPL22 phenotype was previously reported to be absent (4). We confirmed binding of RPL22 to endogenous EBER1 in extracts of an LCL (Fig. 1A). The p53 protein level was determined by Western blotting in LCLs, and the response to treatment using cisplatin, which stabilizes p53, was also tested. There was no significant difference in the p53 levels or responses between the cells with and without EBER1 expression (Fig. 1B), so EBER1 does not appear to affect p53 expression in LCLs.

### Effect of EBERs on cellular miRNAs.

**FIG 2:**
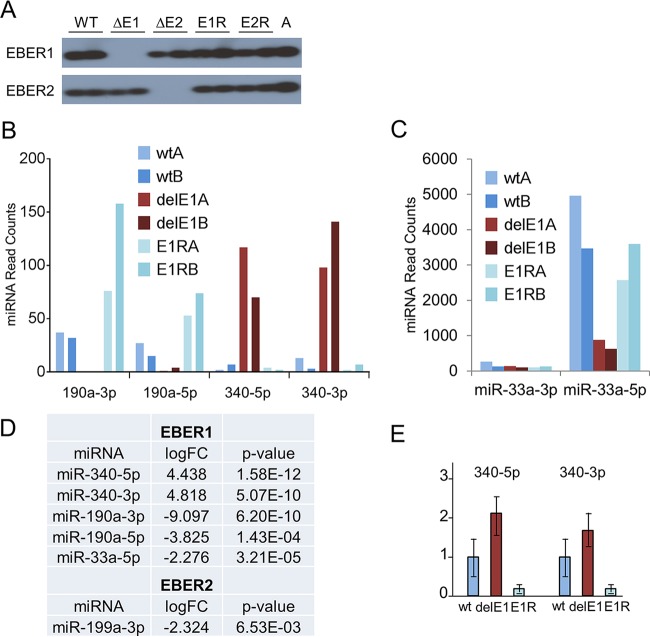
(A) EBER1 and EBER2 expression in the wt and EBER-deleted LCLs used for deep sequencing analysis was confirmed by Northern blotting (1). A 0.5-μg volume of total RNA was used per well. (B) Normalized miRNA read counts for miR-190a and miR-340 in EBER1-deleted (delE1) LCLs compared to wild-type (wt) and EBER1 revertant (E1R) LCLs. “A” and “B” denote the two individual LCLs analyzed for each condition. About 10 million sequencing reads were obtained for each LCL library. Reads were processed as described previously (6) using scripts from the fastx toolkit (http://hannonlab.cshl.edu/fastx_toolkit/). To determine miRNA levels, reads were aligned to the human (HG19) and EBV B95-8 genomes and annotated according to mirbase v21 (http://www.mirbase.org/). To determine miRNA expression levels, miRNA read counts were obtained using the quantifier module of miRDeep2 (14) (see Table S1 in the supplemental material). To identify differentially expressed (DE) miRNAs, miRNA read counts were analyzed by edgeR using trimmed mean of M (TMM) values for normalization (15, 16). For inclusion in the analysis, a minimum of 40 reads per miRNA in at least one of the 10 LCL libraries was required. DE miRNAs with a LogFC *P* value of <0.05 are reported in Table S2. (C) Normalized miRNA read counts for miR-33a in EBER1-deleted LCLs. (D) Summary of log fold changes (logFC) of selected cellular miRNAs showing significant changes correlating with EBER1 or EBER2 expression as determined by deep sequencing and edgeR analysis. (E) Relative levels of miR-340-5p and miR-340-3p determined by TaqMan qRT-PCR assays in LCLs. The expression levels of miRNAs were normalized to RNU48. The values represent means ± standard errors (SE) of the results of analysis of 6 independent samples per EBV type (5 in the case of EBER1 revertants). The wild-type expression levels were set to 1 for each miRNA, whereas the levels of other samples were expressed relative to the wild-type level. Each cDNA sample was analyzed in triplicate.

To determine whether the EBERs might affect cellular microRNAs (miRNAs), we used LCLs containing wild-type (wt) B95-8 bacterial artificial chromosome (BAC) EBV, EBER1 or EBER2 deletion mutants, or revertant viruses. Total RNA was isolated from each LCL, and small RNAs were used to generate Illumina sequencing libraries (6). Appropriate expression of the EBER1 and EBER2 RNAs was confirmed by Northern blotting (Fig. 2A).

Sequencing detected miRNAs from all 8 EBV B95-8 pre-miRNAs and 491 mature human cellular miRNAs. Sequence reads are available at NCBI BioProject PRJNA287267. The general pattern of viral and cellular miRNA expression was comparable to that observed previously in EBV B95-8 LCLs (6); miR-155, miR-146a/b, and miRNAs encoded within the miR-17/92 cluster were highly abundant, and the EBV miRNAs constituted ∼12% of the population (see Table S1 in the supplemental material). As in prior studies (6–8), we found no evidence for production of discrete miRNA-like products from the EBERs.

Sixteen miRNAs (*P* value < 0.01; −2 > log fold change [logFC] > 2) had expression levels altered in response to the deletion of EBER1, while 11 miRNAs had expression levels that were changed in response to EBER2 deletion (see Table S2 in the supplemental material). There were significant differences in the levels of miR-340-3p, miR-340-5p, miR-190a-3p, miR-190a-5p, and miR-33a-5p in EBER1-deleted LCLs compared to wt LCLs (Fig. 2B to D; see also Table S2). Only small changes in levels of expression of miRNAs (such as miR-199a) were observed in EBER2-deleted LCLs (Fig. 2D; see also Table S2). Both the 3p and 5p miRNAs for miR-190 and miR-340 were affected by the deletion of EBER1, indicating that the promoters driving expression of the primary miRNA transcripts were affected (Fig. 2B), consistent with a recent report on miR-190 (9).

The results of additional TaqMan quantitative reverse transcription-PCR (qRT-PCR) assays performed on RNA from independently established LCLs supported the idea of expression of both miR-340-5p and -3p correlating with EBER1 expression (Fig. 2E), but the fold change level was lower in this assay. Since the levels of these miRNAs are all quite low in LCLs (Fig. 2B; see also Table S1 in the supplemental material), we conclude that it is unlikely that the main function of EBERs in LCLs is to alter levels of cellular miRNAs but also that the changes in miRNA expression may contribute to the effects on cellular mRNA levels in LCLs that we reported previously (1).

### Deletion of EBER1 or EBER2 correlates with raised levels of LMP2 RNAs in LCLs.

**FIG 3:**
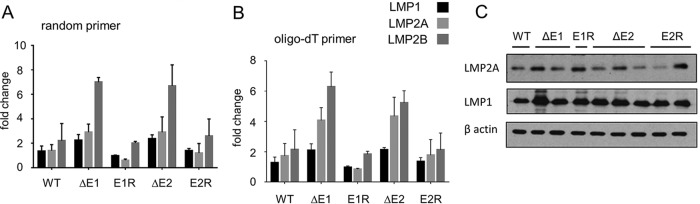
Cytoplasmic RNA from two LCLs each for EBV wild type (WT), deletion of EBER1 (ΔE1), revertant (E1R), and deletion of EBER2 (ΔE2) or revertant (E2R) was used for cDNA synthesis performed with either random primers (A) or oligo(dT) (B) using a ProtoScript First Strand cDNA synthesis kit (New England BioLabs). Quantitative PCR (Q-PCR) performed with the same primers as those described in reference 10 was then used in duplicate assays to measure the levels of RNA for LMP1, LMP2A, and LMP2B, using GAPDH (glyceraldehyde-3-phosphate dehydrogenase) as a reference. The threshold cycle (2^−ΔΔ*CT*^) method of comparative PCR (17) was used to analyze the results, expressed as fold change relative to the E1R LMP1 value. (C) Radioimmunoprecipitation assay (RIPA) lysates were prepared from LCLs, and equal amounts of cell protein were analyzed by Western immunoblotting. Membranes were probed with a 1/1,000 dilution of anti-LMP2A (Abcam; 14B7), a 1/500 dilution of anti-LMP1 (Dako; clone CS.1-4), or a 1/5,000 dilution of anti-β actin (Sigma; AC-74). Secondary antibodies were horseradish peroxidase-conjugated sheep anti-mouse immunoglobulin (GE Healthcare) or horseradish peroxidase-conjugated rabbit anti-rat immunoglobulin (Sigma). Bound immunocomplexes were detected by enhanced chemiluminescence (GE Healthcare).

EBER2 was recently shown to bind PAX5 and to promote PAX5 binding to the terminal repeat region of the EBV genome (10). Knockdown of EBER2 by small interfering (siRNA) caused a 50% increase in LMP1 and LMP2 RNA levels (10). Using quantitative PCR (qPCR) with the same primers on cytoplasmic RNA, we did not observe any significant difference between the EBER2 deletion LCLs and wild-type EBV LCLs in the levels of LMP1 RNA, but LMP2A and LMP2B RNA levels were 2-fold to 3-fold higher when either EBER1 or EBER2 was deleted (Fig. 3A and B), particularly when the cDNA synthesis was primed with oligo(dT) (Fig. 3B). LMP1 and LMP2A protein levels are quite variable in LCLs (Fig. 3C), and there was no apparent correlation with EBER expression. Our results thus provide some support for the idea of EBER2 tending to reduce the level of LMP2 RNA (10), but the specific binding of EBER2 to PAX5 and association with the terminal repeat may not mediate the LMP2 RNA effect that we observed, since there was an increase in the level of LMP2 RNA when either EBER1 or EBER2 was deleted. The focus of the previous study on EBER2 meant that an effect of EBER1 deletion on LMP2 RNA levels was not tested in those experiments (10).

### Infection of mice with a human hematopoietic system with EBV lacking EBER genes.

**FIG 4:**
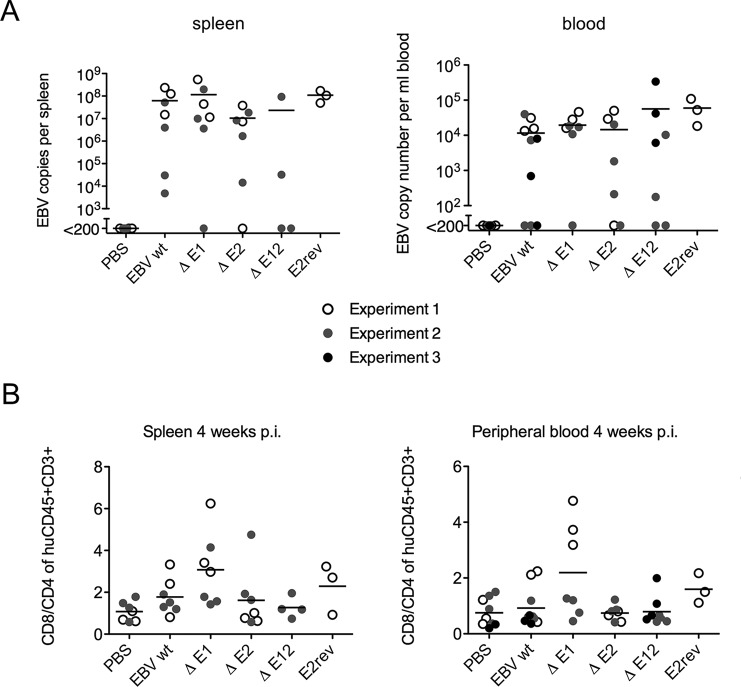
(A) Newborn HLA-A*0201 transgenic NOD/LtSz-Scid IL2RΔnull (NSG-A2tg) mice were irradiated and injected intrahepatically with CD34^+^ human hematopoietic progenitor cells as described previously (18). The reconstitution of human immune system components in the peripheral blood was analyzed prior to the beginning of experiments (normally 12 weeks after engraftment). Groups of mice were infected with 10^5^ infectious units of virus and monitored over a 4-to-8-week period in three experiments, each using different groups of reconstituted mice. EBV loads in spleen and whole blood were quantified 4 weeks after infection for wild-type (EBV wt), EBER1-deficient (EΔ1), EBER2-deficient (EΔ2), EBER1-and-EBER2-deficient (EΔ12), and EBER2 revertant (E2rev) viruses. (B) CD8^+^ T cell expansion was slightly elevated in the absence of EBER1. The T cell ratio of CD8^+^ to CD4^+^ was assessed by flow cytometry after 4 weeks of infection with the same viruses as described for panel A. The composition of blood and spleen samples from the humanized mice was analyzed using anti-human CD45 (HI30; Biolegend), anti-CD3 (UCHT1; Biolegend), anti-CD4 (RPA T4; Biolegend), anti-CD8 (SK1; Biolegend), anti-HLA-DR (L243; Biolegend), anti-CD45RO (UCHL1; BD Pharmingen), and anti-CD19 (HIB19; BD Pharmingen). Spleens were mechanically disrupted and filtered through a 70-μm-pore-size cell strainer. Erythrocytes were lysed in whole blood or in spleen suspensions using NH_4_Cl. Cell suspensions were stained with the indicated antibodies for 15 min at 4°C and washed. Statistical analysis for all mouse experiments used two-tailed *t* tests. A *P* value of <0.05 was considered statistically significant.

Using the EBER mutant EBV strains, we tested whether EBERs affect establishment of EBV infection in NSG mice with a humanized hematopoietic system (11). In three separate experiments involving a total of 44 correctly reconstituted mice, there was no significant difference between the frequencies of infection detected in the blood or in the spleen with deletion of either or both EBER genes from the virus genome. Spleen and blood viral loads are shown in Fig. 4A.

Analyzing parameters of cell-mediated immune responses toward the viral infections, we observed a tendency toward more-pronounced CD8^+^ T cell expansion in the infection with EBER1-deficient viruses (Fig. 4B). However, this tendency was primarily observed in one experiment and was not present in infections with an EBV deficient in both EBERs.

Most studies have focused on cell-intrinsic functions for EBERs, but EBERs have also been reported to be released from cells (12) and in exosomes (13), potentially affecting immune or inflammatory responses (M. Pegtel, personal communication). Most likely, physiological phenotypes for the EBERs will be revealed by *in vivo* infection challenged by normal immune responses. We have now shown that deletion of EBERs does not prevent infection of humanized mice, making this type of analysis possible.

## Supplementary Material

Supplemental material
