# Supplementary material for: Epstein-Barr Viruses (EBVs) Deficient in EBV-Encoded RNAs Have Higher Levels of Latent Membrane Protein 2 RNA Expression in Lymphoblastoid Cell Lines and Efficiently Establish Persistent Infections in Humanized Mice
Source: J Virol. 2015 Sep 2;89(22):11711–4. doi: 10.1128/JVI.01873-15 (PMC4645642; doi:10.1128/JVI.01873-15)
Supplement: Supplemental material [file supp_89_22_11711__index.html]

Supplemental material 

# Epstein-Barr viruses deficient in EBER RNAs give higher LMP2 RNA expression in lymphoblastoid cell lines and efficiently establish persistent infection in humanized mice

## Supplemental material

- Supplemental file 1 -

  Table S1 (miRNA sequencing results.)

  XLSX, 49K
- Supplemental file 2 -

  Table S2 (Differential expression of miRNAs in LCLs with EBER deleted compared to that in wild-type and EBER revertant LCLs.)

  XLSX, 13K
